# Recombination Pattern Reanalysis of Some HIV-1 Circulating Recombination Forms Suggest the Necessity and Difficulty of Revision

**DOI:** 10.1371/journal.pone.0107349

**Published:** 2014-09-09

**Authors:** Lei Jia, Lin Li, Hanping Li, Siyang Liu, Xiaolin Wang, Zuoyi Bao, Tianyi Li, Daomin Zhuang, Yongjian Liu, Jingyun Li

**Affiliations:** Department of AIDS Research, State Key Laboratory of Pathogen and Biosecurity, Beijing Institute of Microbiology and Epidemiology, Fengtai District, Beijing, China; Institute of Infectious Disease and Molecular Medicine, South Africa

## Abstract

**Background:**

Recombination is one of the major mechanisms underlying the generation of HIV-1 variability. Currently 61 circulating recombinant forms of HIV-1 have been identified. With the development of recombination detection techniques and accumulation of HIV-1 reference stains, more accurate mosaic structures of circulating recombinant forms (CRFs), like CRF04 and CRF06, have undergone repeated analysis and upgrades. Such revisions may also be necessary for other CRFs. Unlike previous studies, whose results are based primarily on a single recombination detection program, the current study was based on multiple recombination analysis, which may have produced more impartial results.

**Methods:**

Representative references of 3 categories of intersubtype recombinants were selected, including BC recombinants (CRF07 and CRF08), BG recombinants (CRF23 and CRF24), and BF recombinants (CRF38 and CRF44). They were reanalyzed in detail using both the jumping profile hidden Markov model and RDP3.

**Results:**

The results indicate that revisions and upgrades are very necessary and the entire re-analysis suggested 2 types of revision: (i) length of inserted fragments; and (ii) number of inserted fragments. The reanalysis also indicated that determination of small regions of about 200 bases or fewer should be performed with more caution.

**Conclusion:**

Results indicated that the involvement of multiple recombination detection programs is very necessary. Additionally, results suggested two major challenges, one involving the difficulty of accurately determining the locations of breakpoints and the second involving identification of small regions of about 200 bases or fewer with greater caution. Both indicate the complexity of HIV-1 recombination. The resolution would depend critically on development of a recombination analysis algorithm, accumulation of HIV-1 stains, and a higher sequencing quality. With the changes in recombination pattern, phylogenetic relationships of some CRFs may also change. All these results may be critical to understand the role of recombination in a complex and dynamic HIV evolution.

## Introduction

One of the remarkable characteristics of HIV-1 is the high prevalence of variation. This has produced group M, group N, group O, and the newly described group P [1], [2], [3]. HIV-1 group M is predominant in HIV-1 infections worldwide and can be further divided into nine subtypes (A–D, F–H, J, and K) and five sub-subtypes (A1–A3, F1, and F2). Intersubtype recombination is one major mechanisms contributing to HIV-1 variability, allowing the rapid generation of viral variants with high replicative capacity, drug resistance, and modified expression of antigenic epitopes (summarized in [4], [5]). Analyses of recombination patterns can reveal as much about evolution as analyses of nucleotide substitution patterns do.

Extensive experiments have been performed on HIV-1 and other retroviruses. Results have suggested that HIV-1 genetic recombination is caused by a copy choice mechanism—the alternating use of two templates during the synthesis of a single viral DNA molecule [6]. The recombination of HIV-1 occurs frequently, and can produce many recombinant, currently including 61 HIV-1 circulating recombinant forms (CRFs) and numerous unique recombinant forms (URFs). These CRFs have caused global epidemics. For example, CRF02_AG is common in West Central Africa, CRF07_BC and CRF08_BC are predominant in China [7], [8]. BF is mainly found in South America [9], [10]. A designation of CRF requires three representative strains to be identified in at least three HIV-infected persons without direct epidemiological linkage, and three near full-length genomic (NFLG) sequences are preferred [11]. Almost all published chimeric structures of CRFs have been summarized in the Los Alamos HIV database (http://www.hiv.lanl.gov/content/index).

A more accurate determination of mosaic structure usually requires repeated analysis and upgrades. For example, the strain now designated CRF04_cpx was first identified as the prototype of subtype I based on gp120 sequences [12]. Full genome sequencing revealed this virus to be a complex mosaic with multiple breakpoints between regions of several distinct subtypes, including A, G, and I [13]. Subsequent analysis with previously unavailable complete genome sequences has revealed that the virus is in fact mosaic with regions associated with subtypes A, G, H, and K and unclassified regions [14]. The CRF06 recombinant was previously named “CRF06_AGJ,” but the subsequent identification of subtype K suggested that some regions of CRF06 are subtype K, so the subtype is now designated as “CRF06_cpx” and it includes subtypes A, G, J, and K [15]. The recombinant structure of the reference sequence was subsequently analyzed by Montavon et al. [16]. Similar progress has been made with other CRFs, like CRF13, CRF16, and CRF21 [17], [18], [19]. Considering the development of recombination detection technique and accumulation of appropriate reference sequences, such revisions may also be necessary to make further determinations of recombination patterns for other CRFs. Some of the previously identified CRF mosaic structures are derived completely from Recombination identification program (RIP) (CRF07 and CRF17), some are derived completely from jumping profile hidden Markov model (jpHMM) (CRF52, CRF57, and CRF61), and Simplot was preferred by the majority. Previous results regarding recombination were based primarily on single programs. A more impartial result may be obtained by using more forms of recombination analysis.

In this study, representative references of 3 categories of intersubtype recombinants were selected (a total of 6CRFs), including BC recombinants (CRF07 and CRF08), BG recombinants (CRF23 and CRF24), and BF recombinants (CRF38 and CRF44). Both jpHMM and RDP3 were used to perform a re-analysis of the selected reference strains available from the Los Alamos HIV database [20], [21], [22].

## Materials and Methods

### Sequences

All the reference strains of 6 selected HIV-1 CRFs were extracted from the Los Alamos HIV database. The information of these CRFs and methods of determining original mosaic structure are shown in Table 1. Subtype reference alignments from the Los Alamos HIV database were used to make alignments with the selected strains. Sequences were aligned using muscle implemented in Mega 5 and minor manual adjustments were performed [23].

**Table 1:** Information of 6 reanalyzed CRFs.

Name	Reference strain	Accession number	Methods determining original mosaic structure
CRF07_BC	97CN001_C54	AF286226	RIP
CRF08_BC	97CNGX_6F	AY008715	Simplot
CRF23_BG	CB118	AY900571	Recombinant structures were determined by Simplot. The programs Genconv, MaxChi, and GARD were used to locate more precisely the positions of breakpoints.
CRF24_BG	CB378	AY900574	
CRF38_BF	UY03_3389	FJ213783	Simplot
CRF44_BF	CH80	FJ358521	Simplot

### Recombination detection

jpHMM was first used to perform recombination analysis. This tool is very intelligent and can produce a genome map based directly on HXB2 numbering. This prediction method is based on a precalculated multiple alignment of the major HIV-1 subtypes including CRF01_AE references, and it is more accurate than the competing methods used for phylogenetic breakpoint detection [21]. In jpHMM, each HIV-1 subtype is represented by a profile hidden Markov model. All profile models are connected by empirical probabilities, allowing the detection of possible recombinants and related breakpoints by jumping from one profile to another. jpHMM performs best in predicting recombinants that involve subtypes that have had adequate sampling to build well-informed profiles. It is less effective in cases related to subtypes H, J, and K because so few full-length genome sequences are available (N = 4, 3, and 2, respectively). In the present study, jpHMM was used to detect the recombination patterns in recombinants composed exclusively of subtypes B, C, F, and G. Each of these subtypes has enough data to form a good model of sequence variation. To confirm the data obtained by jpHMM analysis, another recombination analysis tool, RDP3, a software package for statistical identification and characterization of recombination events in DNA sequences, was used to perform further analysis [22]. RDP3 is also very intelligent and simultaneously utilizes a range of non-parametric recombination detection methods: RDP, GENECONV [24] BOOTSCAN [25], [26], MAXCHI [27], [28], CHIMAERA [27], SISCAN [29], and 3SEQ [30]. RDP3 treats every sequence within the analyzed alignment as a potential recombinant and systematically screens sequence triplets or quartets to identify viruses that contain a recombinant and two sequences that could serve as parents while performing a statistical evaluation of recombination signals [22]. Such an approach eliminates the need for reference sequences, which makes analysis of viral quasispecies from epidemiologically unlinked patients more practical [31]. The main strength of RDP3 is that it simultaneously uses a range of different recombination detection methods to both detect and characterize the recombination events that are evident within a sequence alignment without any prior user indication of a non-recombinant set of reference sequences. The sequences are set to linear. The highest acceptable *P*-value is set to 0.05. The other parameters are default RDP3 settings. The HIV-1 sequence would be considered to be recombinant when the recombination signal was supported by at least 3 methods with *P*-values of ≤0.05 after Bonferroni correction for multiple comparisons implemented in RDP3 [22], [32]. The breakpoint position inferred were manually checked using recombination signal analysis implemented in RDP3. Recombinant breakpoint locations were designated relative to HXB2 (Genbank accession no. K03455).

In the current study, when there is a conflict, recombination events detected by at least 2 of the 3 programs (jpHMM, RDP3, and the one determining the original mosaic structure) are considered the actual events. Methods and algorithms of the newly used recombination analysis tools and the original tools are listed in Table 2.

**Table 2:** Lists of methods and algorithms of the four recombination analysis tools.

Program	Method	Implemented algorithms	Publication Year
SimPlot	Phylogenetic methods, Pairwise sequence comparisons	Similarity/distance plot, Bootscanning	1999
RIP	Pairwise sequence comparisons	Similarity/distance plot	1995
jpHMM		Jumping profile hidden Markov models, Hidden Markov models	2009
RDP3	Phylogenetic methods, Patterns of sites, Pairwise sequence comparisons	Sawyer's statistical test for gene conversion, Maximum chi-squared, Similarity/distance plot, Bootscanning, Difference in Sums of Squares method, Graphical recombination detection using Phylogenetic Profiles, Likelihood Analysis of Recombination in DNA, Sister scanning method, RDP method, Automated bootscanning (Recscan), Recombination detection using multiple approaches, Recombination detection using hyper-geometric random walks.	2010

### Phylogenetic analysis

Based on the newly inferred breakpoint locations, fragments with significant conflict were phylogenetically analyzed individually. The phylogenetic tree was constructed using the maximum likelihood method implemented in Mega 5 or using PhyML implemented in RDP3 [23], [33]. The reliability of tree topologies was assessed by bootstrapping using 500 replications. Bootstrap support values of ≥70% were considered significant.

## Results

### Reanalysis of CRF07_BC indicated that there is no subtype B segment insertion in the middle of the gag region

**Figure 1:**
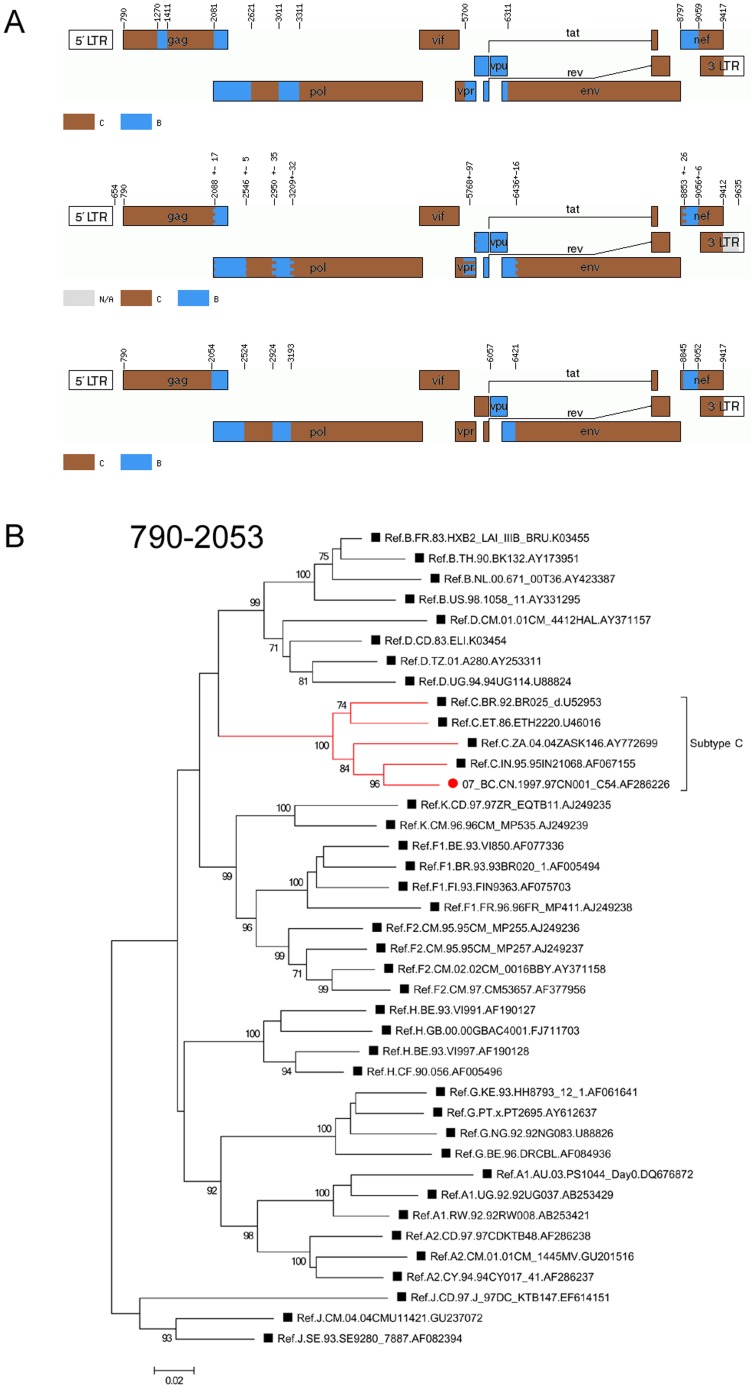
Recombination and phylogenetic reanalysis of CRF07_BC. (A) Genome maps of CRF07_BC from different sources. The top picture shows the original mosaic structure from the Los Alamos HIV database. The middle image is the jpHMM-derived mosaic structure. The bottom image is the RDP3-derived mosaic structure. The standard representatives are marked by different colors, as indicated. (B) Phylogenetic relationship of the region spanning HXB2 nt 790–2053 in gag with the representatives of the major HIV-1 (group M) subtypes based on a new mosaic structure. The tree was constructed using the Maximum likelihood method implemented in Mega 5. Values at the nodes indicate the percent bootstraps in which the cluster to the right was supported. Bootstrap support values of ≥70% were considered significant. Only bootstraps of 70% and higher are shown. Subtype C clades are identified by brackets. Branch lengths are drawn to scale. Positions are shown beside the tree.

Both jpHMM and RDP3 revealed the very similar proposal parents and breakpoint locations of CRF07. An apparent revision is that the originally identified fragment of subtype B in gag gene (HXB2 nt 1270-1410) of CRF07 was, however, not detected (Figure 1A, Table 3). The reanalysis was expanded to other complete sequences of CRF07 in the Los Alamos HIV database and the same results were obtained (data not shown). In order to further confirm the results of the new recombination, this newly inferred recombinant region spanning HXB2 nt 790 to 2053 was used to construct a phylogenetic tree. The tree clearly showed the clustering of the fragment with the C references (Figure 1B) and thus supports the conclusion.

**Table 3:** Comparison of newly identified segment assignment and breakpoint positions of CRF07_BC with original data.

Method of recombination analysis	Segment assignment and breakpoint positions of CRF07_BC				
RIP	B1: 1270 1410	B2: 2081 2620	B3: 3011 3310	B4: 5700 6310	B5: 8797 9058
jphMM	-	2073 2546	2979 3203	5851 6428	8840 9059
RDP3	-	2054 2523	2924 3192	6057 6420	8845 9051
RDP3 plus original reference sequences	-	2059 2546	2914 3185	5670 6431	8869 9059

-indicates that the segment was not detected using this method.

### Reanalysis of CRF08_BC indicated that it has a recombination pattern similar to that of CRF07_BC in the nef region

**Figure 2:**
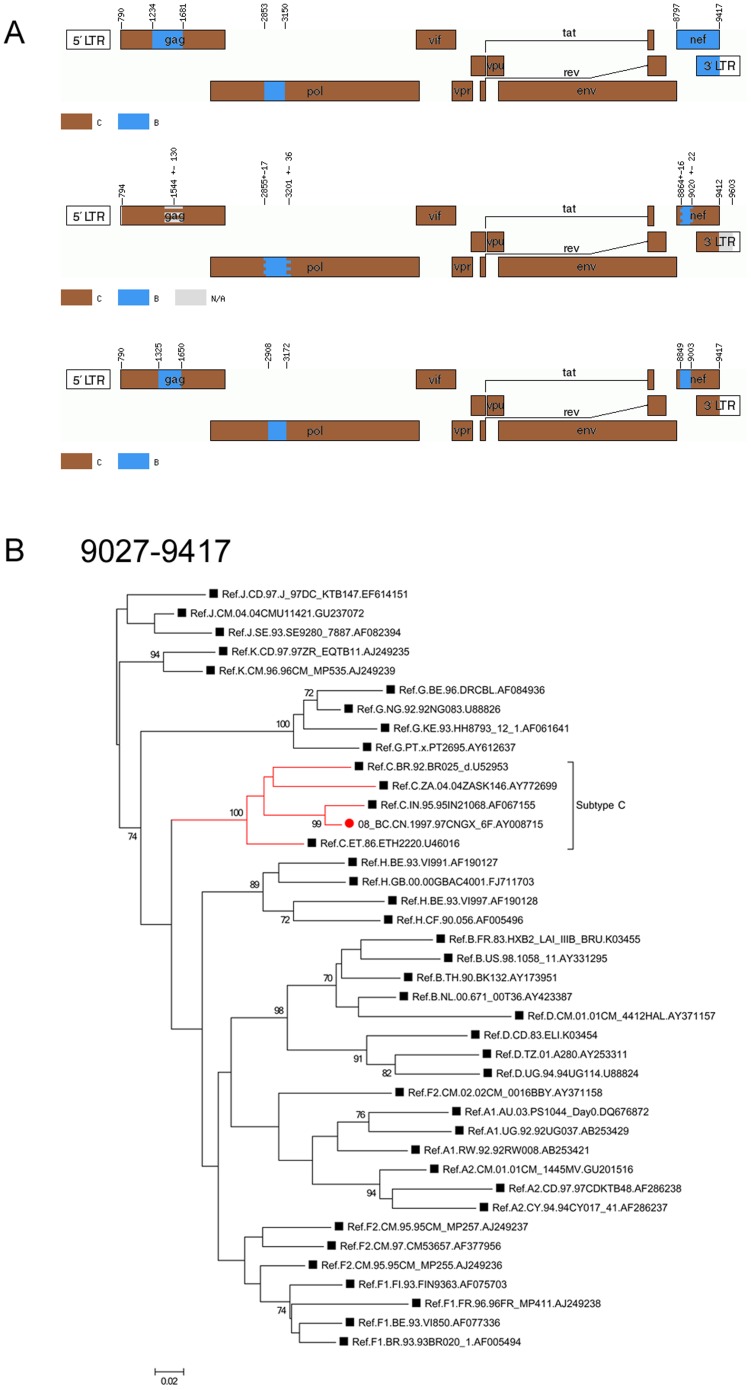
Recombination and phylogenetic reanalysis of CRF08_BC. (A) Genome maps of CRF08_BC from different sources. The first mosaic structure in the panel is from the Los Alamos HIV database. The second is from jpHMM. The third is from RDP3. The standard representatives are marked with different colors, as indicated. (B) Phylogenetic relationship of the region spanning HXB2 nt 9027–9417 in nef with the representatives of the major HIV-1 (group M) subtypes based on newly inferred mosaic structure. The tree was constructed using the maximum likelihood method implemented in Mega 5. Bootstrap support values of ≥70% are considered to be significant.

Unlike CRF07, the first apparent revision found in CRF08 lies in that B segment originally spanning HXB2 nt 8797–9417 in the nef gene was reduced to HXB2 nt 8864–9026 by jpHMM and 8849–9002 by RDP3 (Figure 2A, Table 4), thus exhibiting a very similar recombination pattern to that of CRF07_BC. The remaining region of nef was reclassified as a C subtype. This newly inferred result was confirmed by Maximum Likelihood trees constructed using Mega 5. The results clearly indicate the clustering of the conflicted region together with the C reference strains (Figure 2B). The redetermination of recombination patterns is critical to understanding the phylogenetic relationship between different recombinants, because the interpretation of phylogenetic relationships depends critically on a more accurate genome map of CRFs. For example, the results of reanalysis of CRF07 and CRF08 in the nef region indicated a much closer phylogenetic relationship between these CRFs than had previously been believed (Figures 1A and 2A).

**Table 4:** Comparison of newly identified segment assignment and breakpoint positions of CRF08_BC with original data.

Method of recombination analysis	Segment assignment and breakpoint positions of CRF08_BC		
Simplot	B1: 1234 1680	B2: 2853 3149	B3: 8797 9417
jphMM	-	2842 3177	8864 9026
RDP3	1325 1649	2908 3171	8849 9002
RDP3 plus original reference sequences	1268 1641	2866 3171	8869 9028

-indicates that the segment was not detected using this method.

With respect to the previously characterized B segment spanning HXB2 nt 1234–1680 in gag, RDP3 detected a little shorter B insertion spanning HXB2 nt 1325–1649, but it was re-identified as an uncertainty region (HXB2 nt 1414–1674) in jpHMM-derived results. The phylogenetic tree of the region spanning HXB2 nt 1325–1649 indicates that there indeed is a B segment (data not shown). Unlike the alignment in the RDP3 and in original Simplot, in jpHMM, each HIV-1 subtype is represented by a profile hidden Markov model. This difference may be a reason for the conflicted results by jpHMM.

To further address the presence of new different recombination forms, the reference sequence of subtype B' in original literatures of CRF07 and CRF08, RL42 from Yunnan, China (GenBank accession number U71182), was added to the subtype reference alignments to re-perform RDP analysis (The reference sequences of subtype C used in the bootscanning analysis of CRF07 and CRF08 in original literatures is eth2220 and 95IN21068, respectively [34], [35]. Both strains have been included in the subtype reference alignments.). The results are shown in Table 3 (CRF07) and Table 4 (CRF08). Both show very similar results to that of the first round of analysis. For example, there is no subtype B insertion in middle region of gag of CRF07 and the original larger B fragment in nef region of CRF08 is modified to a pattern very similar to that of CRF07.

### Reanalysis of CRF24_BG and CRF44_BF indicate that revisions are necessary

**Figure 3:**
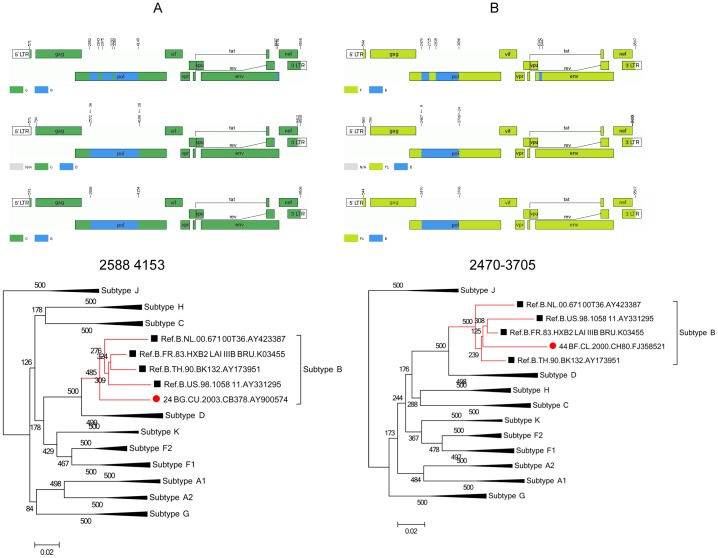
Recombination and phylogenetic reanalysis of CRF24_BG and CRF44_BF. (A) Results of CRF24 from both jpHMM and RDP3 indicate there is a whole B segment in the pol region rather than three separate segments in the previous structure. The result was further confirmed by the phylogenetic tree. The position spans 2588–4153 is shown above the tree. The tree was constructed using PhyML program implemented in the RDP3 software package. Bootstrap replications of ≥350 (i.e., bootstrap support values of ≥70%) are considered to be significant. The very small part of the B segment (8697–8750) in env was not detected by either newly-used program. (B) Both jpHMM and RDP3 indicate that in CRF44, there is a whole B segment in the pol region rather than two separate segments in previous mosaic structure. The result was further confirmed by the phylogenetic analysis. The position spans 2470–3705 is shown above the tree. The tree was constructed using PhyML program implemented in the RDP3 software package. Bootstrap replications of ≥350 (i.e., bootstrap support values of ≥70%) are considered to be significant. A very small part of B segment (6342–6446) in env was not detected by either new program.

The results of reanalysis of CRF24 and CRF44 are summarized in Figure 3. The contents of both CRFs found are consistent with original ones. According to new schemes, the first apparent difference of CRF24 lies in region spanning 2552–4148. The original structure indicates that it is an interval of 3 B segments and 2 G segments. Both jpHMM and RDP3 revealed that it is a complete pure region of subtype B (details see Figure 3A and Table 5). Phylogenetic analysis further confirmed this (Figure 3A). The original very small B segment spanning 8697–8750 in env of 54 bases was not detected by either of these two programs. With short genes of less than 200 bases, a reliable tree cannot be produced so no phylogenetic tree was provided for this region. Similarly, a complete larger B segment was found in pol according to new schemes of CRF44 rather than the original interval of 2 B segments and 1 F1 segment (Figure 3B and Table 6). The subsequent phylogenetic analysis of HXB2 nt 2470–3705 supported these results closely. In addition, the originally characterized small insertion of subtype B segment spanning 6342–6446 (105 bases) in env was not found by either jpHMM or RDP3. In this way, both CRF24 and CRF44 present a clearer pattern of mosaicism than had previously been believed.

**Table 5:** Comparison of newly identified segment assignment and breakpoint positions of CRF24_BG with original data.

Method of recombination analysis	Segment assignment and breakpoint positions of CRF24_BG			
Simplot	B1: 2552 2842	B2: 2975 3292	B3: 3380 4148	B4:8697 8750
jphMM	2574 4208			-
RDP3	2588 4153			-
RDP3 plus original reference sequences	2591 4153			-

-indicates that the segment was not detected using this method.

**Table 6:** Comparison of newly identified segment assignment and breakpoint positions of CRF44_BF with original data.

Method of recombination analysis	Segment assignment and breakpoint positions of CRF44_BF		
Simplot	B1: 2470 2724	B2: 2935 3695	B3: 6342 6446
jphMM	2465 3729		-
RDP3	2470 3705		-

-indicates that the segment was not detected using this method.

In conclusion, reanalysis results of CRF07, CRF08, CRF24, and CRF44 suggest that the revisions mainly include 2 categories: (i) length of inserted fragments; and (ii) number of inserted fragments.

### Reanalysis of CRF23_BG and CRF38_BF indicate that recombination analysis is difficult

**Figure 4:**
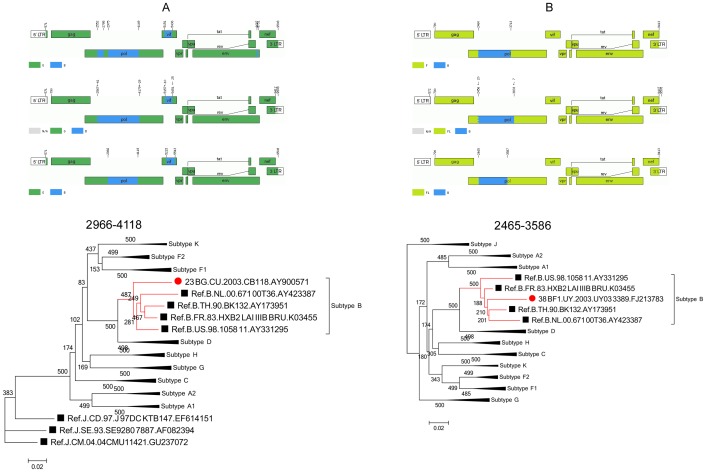
Reanalysis of CRF23_BG and CRF38_BF reveal the challenges of recombination analysis. (A) Three recombination programs indicate similar endpoints but significantly different starting points of the B segment in pol region of CRF23. (B) These three recombination programs indicate similar starting points but different endpoints of the B segment in pol region of CRF38.

Reanalysis of CRF23_BG and CRF38_BF produced impenetrable results and suggests the difficulty of identifying recombination patterns. As shown in Figure 4A and Table S1, all 3 recombination programs indicate similar endpoints but different starting points of the B segment in pol of CRF23. The jpHMM-derived result indicates that the starting point was 2567. The RDP3-derived result indicates that the starting point is 2966. The starting point in original data set was 2552 with an additional G segment spanning 2795–2974 of 180 bases. As shown in Figure 4B and Table S2, all 3 recombination programs indicate similar starting point but different end point of the B segment in pol of CRF38. The endpoint in the original data set was 3712. The jpHMM-derived result indicated that the endpoint was 3832, 120 bases longer than original result. The RDP3-derived result indicated that the endpoint was 3586, 126 bases shorter than original result. These results raised the first challenge that is the difficulty of locating the breakpoint accurately during recombination pattern analysis. The challenge also held true for the other 4 CRFs. Different programs always produced different breakpoint locations, even when the patterns were very similar. In addition, there was also a second challenge. The reanalysis indicated that determination of small regions of about 200 bases or fewer must be performed with caution. For example, previous results of subtype B insertion in gag region of CRF07 (141 bases), subtype B insertion in env regions of CRF23 and CRF24 (54 bases), subtype B insertion in env region of CRF44 (105 bases) were not all indicated by both jpHMM and RDP3. It is well known that phylogenetic tree analysis is a very general method used to confirm the recombination mosaic structures. However, this confirmation depends critically on the mosaic structure derived from the results of recombination tools such as Simplot, jpHMM, RIP, and RDP. In other words, no segment can be further confirmed using phylogenetic tree analysis unless it is first indicated to be imbedded in wild virus sequence by a recombination tool,. As an example of CRF07, in the current of re-analysis, neither of newly used methods based on different background alignments found any fragments of subtype B in the middle region of gag of CRF07. Thus no subtype B fragment can be further confirmed using phylogenetic analysis. Additionally, as has been discussed by Leitner et al., such small regions contain too little sequence information to produce reliable phylogenetic trees (http://www.hiv.lanl.gov/content/sequence/HIV/REVIEWS/RefSeqs2005/RefSeqs05.html). Therefore, direct subtype re-assignment of the originally identified small B segment in CRF07 based on construction of a phylogenetic tree is unreliable. To better describe the issue, a maximum likelihood-based tree was constructed for the subtype B insertion in gag region of CRF07 spanning HXB2 nt 1270-1410 (141 bases). The unreliable tree involved both incorrect clades for the subtype references and very low bootstrap values (data not shown). Both challenges described above indicated the complexity of HIV-1 recombination. It is here suggested that the involvement of multiple recombination detection programs may facilitate the production of impartial results.

In original literatures, both phylogenetic and bootscanning analysis of CRF38 and CRF44 were also based on the subtype reference alignments. While bootscanning analysis of CRF23 and CRF24 were based on locally circulating strains, subtype B sequence of Cu19 (Accession number AY586542) and subtype G sequences of Cu74 (Accession number AY586547) [36]. Therefore, additional recombination analysis were performed using the subtype reference alignments plus these two strains for CRF23 and CRF24. The results showed that proposal parents of both CRFs were more closely related to this two locally circulating stains. Meanwhile, the results clearly indicated a very similar presence of new different recombination forms as the first round of analysis did, compared to the originally named CFRs in Los Alamos HIV-1 sequence database (Table 5 and Table S1).

## Discussion

With the development of a variety of tools for the detection of recombinant genomes and accumulation of HIV-1 reference stains, more accurate mosaic structures of CRFs, like CRF04 and CRF06, have undergone repeated analyses and upgrades. The present work included a large-scale reanalysis of 6 CRFs. Both the recombination analysis and subsequent phylogenetic analysis indicated that such revisions and upgrades are very necessary. In total, 2 types of revisions were made. It will become increasingly valuable to know, in full phylogeny detail, the circulating recombinant form of HIV-1, because the interpretation of phylogenetic relationships of different recombinants (including confirmation of mosaic structure by sub-region trees) depends critically on this information.

The present study is the first to use RDP3 to perform recombination patterns analysis of HIV-1 CRFs. The main strength of RDP3 is that it simultaneously uses a range of different recombination detection methods to both detect and characterize the recombination events. It is more likely than other methods to produce impartial results. In addition to the algorithm, background alignments are also very important to recombination analysis. The jpHMM method used in this study is based on a precalculated multiple alignment of the major HIV-1 subtypes, including CRF01_AE references. Well-informed profiles are built using adequate sampling of each subtype. Then each HIV-1 subtype is represented by a profile hidden Markov model. In this way, at least from these two points, the newly derived results can reveal recombination patterns better than other methods can.

These results illustrate two major challenges. One involves the difficulty of determining the location of the breakpoint accurately. The second is that determination of small regions about 200 bases or fewer should be performed with caution. During the current work, unreliable trees were found and contained both incorrect clades of subtype references and very low bootstrap values (data not shown). Both challenges indicated the complexity of HIV-1 recombination. The resolution was found to depend critically on development of recombination analysis algorithm, accumulation of HIV-1 stains (which can lead to more representative background alignments), and a higher quality of sequencing.

## Supporting Information

Table S1 Comparison of newly identified segment assignment and breakpoint positions of CRF23_BG with original data.
(DOCX)

Table S2 Comparison of newly identified segment assignment and breakpoint positions of CRF38_BF with original data.
(DOCX)
