# Correction: Recombination Pattern Reanalysis of Some HIV-1 Circulating Recombination Forms Suggest the Necessity and Difficulty of Revision

**DOI:** 10.1371/journal.pone.0155750

**Published:** 2016-05-11

**Authors:** Lei Jia, Lin Li, Hanping Li, Siyang Liu, Xiaolin Wang, Zuoyi Bao, Tianyi Li, Daomin Zhuang, Yongjian Liu, Jingyun Li, The PLOS ONE Editors

It was brought to the attention of *PLOS ONE* staff editors that there are similarities in study design and some findings between this article and the following previously published work:

Zhang M, Foley B, Schultz AK, Macke JP, Bulla I, Stanke M, Morgenstern B, Korber B, Leitner T: The role of recombination in the emergence of a complex and dynamic HIV epidemic. Retrovirology 2010 Mar, 7:25. doi:10.1186/1742-4690-7-25 [2]

This previously published study used the jpHMM program to analyze sequences, including BC recombinants CRF07 and CRF08 and BF recombinants, and thus reported work related to the findings described in the current publication.

Jia et al. have used the same program, jpHMM, as well as RDP3, to analyze HIV sequences. The authors wish to clarify that the current article was focused on describing a more accurate recombination pattern of the Reference strains of 6 selected circulating recombinant forms (CRFs) compared to those previously reported, while Zhang et al. reported whether full-length and sequence fragments (but not the Reference strains) of CRFs required subtype assignment. Jia et al. also discuss a comparison between jpHMM-derived recombination structures of Reference strains of the CRFs with the original ones listed on the HIV Circulating Recombinant Forms page of the Los Alamos database (http://www.hiv.lanl.gov/content/sequence/HIV/CRFs/CRFs.html).

In addition, the following paragraph from the Methods section was duplicated almost verbatim from the above article [2]:

“In jpHMM, each HIV-1 subtype is represented by a profile hidden Markov model. All profile models are connected by empirical probabilities, allowing the detection of possible recombinants and related breakpoints by jumping from one profile to another. jpHMM performs best in predicting recombinants that involve subtypes that have had adequate sampling to build well-informed profiles. It is less effective in cases related to subtypes H, J, and K because so few full-length genome sequences are available (N = 4, 3, and 2, respectively). In the present study, jpHMM was used to detect the recombination patterns in recombinants composed exclusively of subtypes B, C, F, and G. Each of these subtypes has enough data to form a good model of sequence variation.”

The authors wished to ensure an accurate description of the jpHMM tool by using the description previously published by the developers of the tool; however, the authors recognize the sentences should have been reproduced in quotation marks.

Figure 2B of the published article shows some similar findings to those presented in the following thesis, which was not cited:

Doctoral Thesis Ming Zhang: Tracking HIV-1 Genetic Variation https://www.uni-goettingen.de/en/zhang-ming-tracking-hiv-1-genetic-variation/73586.html [3]

Similarly to the work on the thesis, Figure 2B in this article displays sub-regions trees; however, the authors would like to clarify that the goal is to confirm the subtype adscription of the fragment in nef region of CRF08, while the thesis reports trees constructed for sub-regions delimited by all CRF07 and CRF08 sequences in order to investigate the relationship between CRF07 and CRF08.

Additional instances of duplicated text were also identified within the following excerpts from the Introduction and Methods sections:

“RDP3 treats every sequence within the analyzed alignment as a potential recombinant and systematically screens sequence triplets or quartets to identify viruses that contain a recombinant and two sequences that could serve as parents while performing a statistical evaluation of recombination signals … Such an approach eliminates the need for reference sequences, which makes analysis of viral quasispecies from epidemiologically unlinked patients more practical.”

The above text is similar to that found in “Frequent Intra-Subtype Recombination among HIV-1 Circulating in Tanzania” *PLOS ONE* DOI: 10.1371/journal.pone.0071131. [4]

"as the prototype of subtype I based on gp120 sequences … Full genome sequencing revealed this virus to be a complex mosaic with multiple breakpoints between regions of several distinct subtypes, including A, G, and I … Subsequent analysis with previously unavailable complete genome sequences has revealed that the virus is in fact mosaic with regions associated with subtypes A, G, H, and K and unclassified regions."

The above text is similar to that found in “CRF06-cpx: A New Circulating Recombinant Form of HIV-1 in West Africa Involving Subtypes A, G, K, and J.” *JAIDS Journal of Acquired Immune Deficiency Syndromes* 29:522–530. [5]

"This prediction method is based on a precalculated multiple alignment of the major HIV-1 subtypes including CRF01_AE references, and it is more accurate than the competing methods used for phylogenetic breakpoint detection."

The above text is similar to that found in “Identification and Genetic Characterization of a Novel CRF22_01A1 Recombinant Form of HIV Type 1 in Cameroon” DOI:10.1089/aid.2009.0197. [6]

The authors would like to apologize for all instances of overlapping text.

## References

[1] Jia L, Li L, Li H, Liu S, Wang X, Bao Z, et al (2014) Recombination Pattern Reanalysis of Some HIV-1 Circulating Recombination Forms Suggest the Necessity and Difficulty of Revision. PLoS ONE 9(9): e107349 10.1371/journal.pone.0107349 25203725 PMC4159329

[2] Zhang M, Foley B, Schultz AK, Macke JP, Bulla I, Stanke M, et al: The role of recombination in the emergence of a complex and dynamic HIV epidemic. Retrovirology 2010 3, 7:25 10.1186/1742-4690-7-25 20331894 PMC2855530

[3] Zhang M: Tracking HIV-1 Genetic Variation. Doctoral Thesis 2007. Available: https://www.uni-goettingen.de/en/zhang-ming-tracking-hiv-1-genetic-variation/73586.html

[4] Kiwelu IE, Novitsky V, Margolin L, Baca J, Manongi R, Sam N, et al Frequent Intra-Subtype Recombination among HIV-1 Circulating in Tanzania. PLOS ONE. 10.1371/journal.pone.0071131 PMC3733632 23940702

[5] Montavon C, Toure-Kane C, Nkengasong JN, Vergne L, Hertogs K, Mboup S, et al CRF06-cpx: A New Circulating Recombinant Form of HIV-1 in West Africa Involving Subtypes A, G, K, and J. J Acquir Immune Defic Syndr. 2002; 29:522–530. 11981370 10.1097/00126334-200204150-00014

[6] Zhao J, Tang S, Ragupathy V, Carr JK, Wolfe ND, Awazi B, et al Identification and Genetic Characterization of a Novel CRF22_01A1 Recombinant Form of HIV Type 1 in Cameroon 10.1089/aid.2009.0197 PMC2931544 20812894

