# Supplementary material for: Recombination Pattern Reanalysis of Some HIV-1 Circulating Recombination Forms Suggest the Necessity and Difficulty of Revision
Source: PLoS One. 2014 Sep 9;9(9):e107349. doi: 10.1371/journal.pone.0107349 (PMC4159329; doi:10.1371/journal.pone.0107349)
Supplement: Table S1 — Comparison of newly identified segment assignment and breakpoint positions of CRF23_BG with original data. (DOCX) [file pone.0107349.s001.docx]

**Table S1.** Comparison of newly identified segment assignment and breakpoint positions of CRF23_BG with original data.

| Method of recombination analysis | Segment assignment and breakpoint positions of CRF23_BG | | | |
| --- | --- | --- | --- | --- |
| Simplot | B1: 2552 2794 | B2: 2975 4148 | B3: 5151 5435 | B4: 8697 8750 |
| jphMM | 2553 4156 | | 5156 5459 | - |
| RDP3 | - | 2966 4118 | 5215 5542 | - |
| RDP3 plus original sequences | - | 2983 4160 | 5150 5446 | - |

- indicates the non-detection of the segment using this method.
