# Supplementary material for: Recombination Pattern Reanalysis of Some HIV-1 Circulating Recombination Forms Suggest the Necessity and Difficulty of Revision
Source: PLoS One. 2014 Sep 9;9(9):e107349. doi: 10.1371/journal.pone.0107349 (PMC4159329; doi:10.1371/journal.pone.0107349)
Supplement: Table S2 — Comparison of newly identified segment assignment and breakpoint positions of CRF38_BF with original data. (DOCX) [file pone.0107349.s002.docx]

**Table S2.** Comparison of newly identified segment assignment and breakpoint positions of CRF38_BF with original data.

| Method of recombination analysis | Segment assignment and breakpoint positions of CRF38_BF |
| --- | --- |
| Simplot | B1: 2460 3712 |
| jphMM | 2468 3838 |
| RDP3 | 2465 3586 |
